# Probiotic and Postbiotic Delivery Systems for the Management of Chronic Wounds: A Review of Emerging Strategies

**DOI:** 10.7759/cureus.105081

**Published:** 2026-03-12

**Authors:** Maja Karmińska, Krystian Czernikiewicz, Wiktoria Głowacka-Kamińska, Natalia Skrzypska, Justyna Wróblewska, Olga Wojtczak, Jakub Tarczykowski, Kacper Zagaja

**Affiliations:** 1 General Medicine, Wojewódzki Szpital Wielospecjalistyczny im. dr. Jana Jonstona w Lesznie, Leszno, POL; 2 Internal Medicine, Prof. S. T. Dabrowski Hospital in Puszczykowo, Poznan, POL; 3 General Practice, F. Raszeja Municipal Hospital, Poznan, POL; 4 Internal Medicine, Hipolit Cegielski Poznan (HCP) Medical Centre, Poznan, POL; 5 Hospital Medicine, University Clinical Hospital in Poznan, Poznan, POL; 6 General Practice, Uniwersytecki Szpital Kliniczny w Poznaniu, Poznan, POL

**Keywords:** burn wound, chronic wound, diabetic ulcer, dressings, lactobacillus plantarum, probiotics, wound healing

## Abstract

Chronic wounds, particularly diabetic foot ulcers (DFUs) and burn wounds (BWs), impact millions of individuals globally and generate substantial healthcare costs, highlighting the urgent need for more effective therapies aimed at controlling infections. Currently, there is growing evidence of beneficial effects of selected bacterial strains, including *Lactobacillus plantarum*, *Lactobacillus reuteri*, *Lactobacillus rhamnosus*, *Lactobacillus paracasei*, *Bifidobacterium longum*, and *Escherichia coli*, as well as fungi from the genus *Saccharomyces* spp. Each of these species exerts a distinct influence on the wound microenvironment. This review compiles evidence from PubMed-indexed studies published between 2016 and 2026 regarding the application of external dressings and other topically administered formulations containing probiotic or postbiotic preparations in the management of DFUs and BWs.

Probiotic strains facilitate wound healing through various mechanisms, including the secretion of antimicrobial metabolites (such as organic acids, hydrogen peroxide, and bacteriocins), inhibition of biofilm formation by pathogens like *Staphylococcus aureus* and *Pseudomonas aeruginosa*, immunomodulation, local acidification, and stimulation of growth factors and components of the extracellular matrix. Various methods for delivering microorganisms with probiotic properties are being evaluated. These approaches can be broadly divided into two main categories: probiotic-containing dressings and non-dressing-based methods (i.e., ointments or biohybrid microneedles). It is indicated that postbiotics - metabolic byproducts or structural components of microbial cells - may likewise contribute positively to wound repair.

A reduction in pathogen load and positive healing outcomes in DFUs and venous or burn ulcers with *L. plantarum* dressings is indicated, although evidence sizes are still limited. Current evidence suggests that probiotic-based dressings could serve as a promising adjunctive strategy in the management of chronic wounds; however, further well-designed clinical trials are necessary to validate efficacy, identify optimal strains and carriers, and assess long-term safety. While a large body of in vitro and animal model data exists, robust evidence from randomized controlled trials in humans remains scarce.

## Introduction and background

Skin is the largest human organ, accounting for approximately 15% of total body weight, and constitutes the body’s first line of defense against environmental factors. It consists of two main layers: the epidermis and the dermis [1,2]. The epidermis protects against pathogens and environmental damage and ensures structural integrity. It is composed mainly of keratinocytes, dendritic cells, melanocytes, Merkel cells, Langerhans cells, and various populations of stem cells [2,3].

The dermis is a thicker layer composed mainly of fibroblasts and a collagen-rich extracellular matrix (ECM), as well as vascular and mechanoreceptors. Both layers contain immune cells that play a key role in monitoring and maintaining skin homeostasis [3,4].

Disruption of the skin barrier damages the surface and supporting structures and allows microorganisms to migrate from the skin surface into the underlying tissues, leading to colonization. Pathogenic microbiota play a negative role in the wound healing process [5,6]. The main components of the microbiota include *Staphylococcus*, *Pseudomonas*, *Corynebacterium*, *Streptococcus*, and *Enterococcus* species [7,8].

Depending on their causes and effects, wounds are classified as acute or chronic. Chronic wounds are defined as non-healing lesions that persist for more than three months or recur after initial closure. They may result from damage to all layers of the skin, including the epidermis, dermis, and subcutaneous tissue [9,10]. Chronic wounds are commonly colonized by bacteria capable of forming biofilms - complex, protective glycocalyx structures - which delay healing and complicate the treatment of wound infections [5,9].

Chronic wounds represent a growing global health problem, affecting an estimated millions of people worldwide and imposing a substantial financial burden on healthcare systems. In the United States alone, chronic wounds affect approximately 6.5 million people annually, and the cost of treating them is estimated to reach up to $25 billion (USD) each year [11]. Factors that contribute to delayed wound healing and the progression to chronic wounds include diabetes, whose prevalence is rising, vascular insufficiency, nutritional deficiencies, neurological disorders, infections, and aging. Consequently, research into wound healing has gained increasing importance in recent years [4,9,10].

This article will discuss and serve as an approach to systematizing the most recent available knowledge about the use of external dressings and other topically applied formulations containing probiotic species, whose supportive effects in wound treatment have been observed and described in numerous publications. Recently, this topic has garnered increasing interest from researchers. Various articles have reported promising benefits from the use of dressings that include strains of *Lactobacillus*, particularly *Lactobacillus plantarum*. The study's aim is to highlight innovative methods concerning probiotics in the treatment of diabetic foot ulcers (DFUs) and burn wounds (BWs).

## Review

Materials and methods

**Figure 1:**
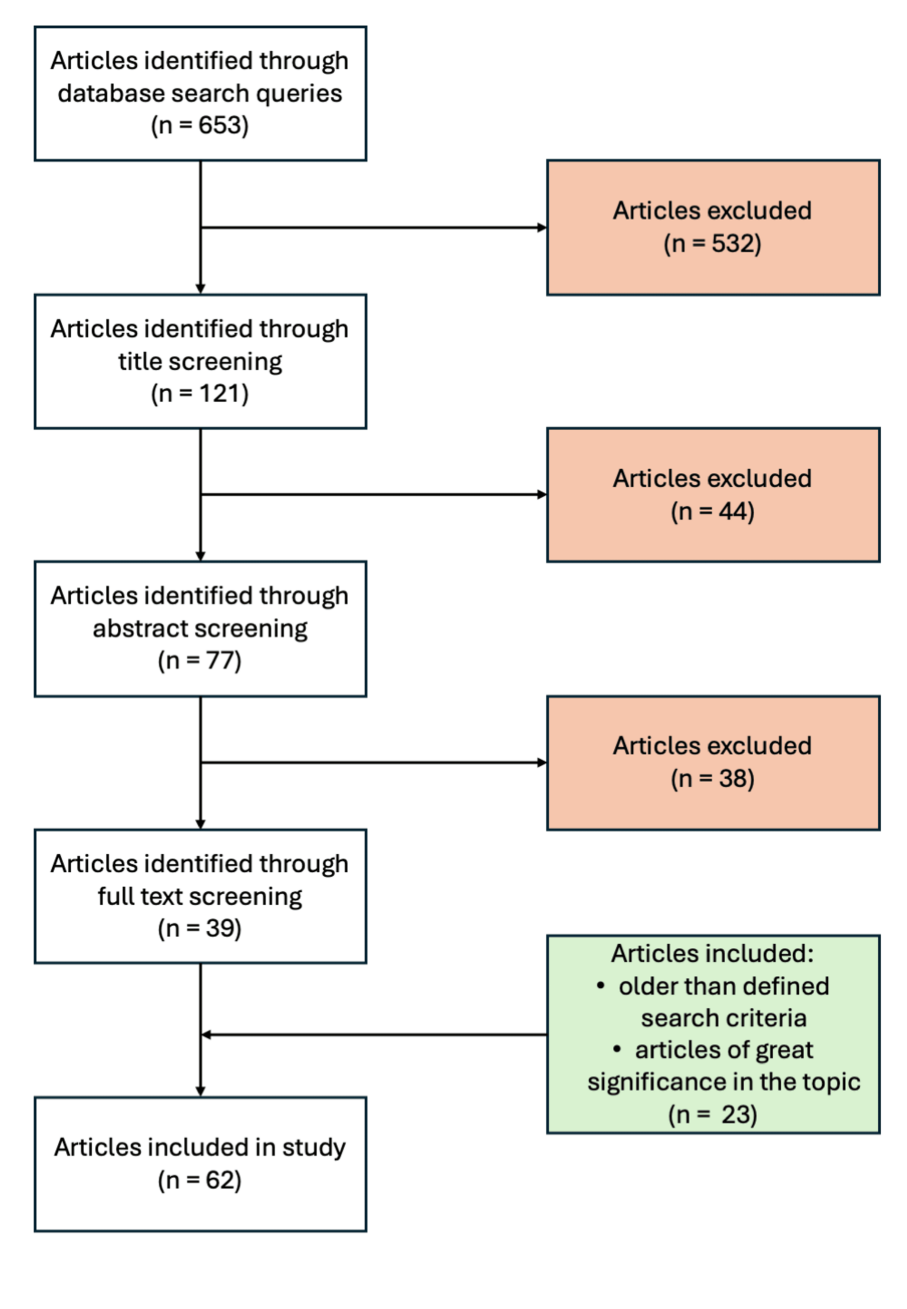
Graphical presentation of the search strategy used in this review.

A thorough search of publicly available PubMed databases was conducted to identify articles relevant to the review topic. The search was limited to publications from 2016 to 2026 in order to provide the most up-to-date evidence from the past decade. The search strategy was based on two main search strings: ("Wound Healing"[Mesh] OR "Wounds and Injuries"[Mesh] OR "Surgical Wound"[Mesh] OR "Surgical Wound Infection"[Mesh] OR "Skin Ulcer"[Mesh] OR "Pressure Ulcer"[Mesh] OR "Diabetic Foot"[Mesh] OR "Burns"[Mesh])AND("Probiotics"[Mesh] OR "Lactobacillus"[Mesh] OR "Bifidobacterium"[Mesh] OR "Saccharomyces"[Mesh] OR "Synbiotics"[Mesh] OR probiotic*[tiab] OR synbiotic*[tiab] OR lactobacill*[tiab] OR bifidobacter*[tiab] OR "Saccharomyces boulardii"[tiab]) and (("Hydrogels"[Mesh] OR hydrogel* OR "hydrogel dressing*" OR "wound hydrogel") AND ("Probiotics"[Mesh] OR probiotic* OR "probiotic bacteria" OR "live probiotic*" OR "viable probiotic*" OR Lactobacillus OR Bifidobacterium OR "Lactic Acid Bacteria" OR LAB) AND ("Wound Healing"[Mesh] OR "wound healing" OR "wound repair" OR "skin regeneration" OR "cutaneous wound") AND ("Bandages"[Mesh] OR dressing* OR "wound dressing" OR "bioactive dressing" OR "therapeutic dressing")) NOT (review[Publication Type] OR "drug delivery" OR nanoparticle* OR "antibacterial hydrogel" OR "antimicrobial hydrogel"). This approach was designed to maximize the number of results and provide a comprehensive literature search. Studies were included in the review if they investigated live probiotic microorganism inactivated preparations, or probiotic-derived products (postbiotics), evaluated their application in chronic wound models, and were conducted in human subjects, animal models, or relevant in vitro systems. Studies were excluded if they assessed microbiological interventions unrelated to probiotics or postbiotics, or were written in a language other than English. All types of articles meeting the inclusion criteria were considered. The articles were reviewed based on their titles and abstracts to select the pool of studies for full analysis. Only articles written in English were included. Additionally, works that did not strictly meet search criteria were incorporated into the review due to their significant contribution to knowledge, key findings, or mechanisms essential for understanding the topic. This strategy for conducting the literature review is illustrated in Figure 1.

Description of wound types encompassed by the study

The selected publications for this research primarily focus on chronic wounds, specifically differentiating between diabetic ulcers and BWs. However, some studies also address the healing of overall skin wounds. This paragraph outlines the pathological processes that contribute to the formation of these wounds, the potential for subsequent infections, and the impact these factors have on healing prognosis as the wounds develop.

Wound healing process

Wound healing is paramount for maintaining quality of life, as it enables the restoration of anatomical and functional integrity and thereby supports the maintenance of homeostasis [4,12]. It is a complex, multistep process comprising four overlapping phases: hemostasis, inflammation, proliferation, and remodeling [13].

The hemostatic phase begins within seconds after injury and prevents blood loss through vasoconstriction and the formation of a fibrin clot initiated by platelet aggregation. Activated platelets subsequently release a variety of mediators that recruit neutrophils, macrophages, and fibroblasts to the site of injury [4,12,14,15]. As a result of activation of the coagulation and complement cascades, vasoactive mediators such as bradykinin and the anaphylatoxins C3a and C5a are generated. These mediators increase vascular permeability, facilitating the extravasation of neutrophils and monocytes into the wound area [4,15].

During the inflammatory phase, neutrophils are the first cells to infiltrate the wound site. They release antimicrobial peptides, proteases, and reactive oxygen species (ROS), which are essential for bacterial clearance [13,15,16]. In the absence of sustained inflammatory signals, neutrophils undergo spontaneous apoptosis mediated by caspase-dependent pathways and cathepsin D. Prolonged persistence of neutrophils in the wound may contribute to the development of chronic, non-healing wounds [4,15,16]. Monocytes become the predominant inflammatory cell population within the wound 2-3 days after injury. They are recruited to the wound through chemotaxis mediated by the chemokine CCL2 and subsequently differentiate into macrophages. Macrophages play a central role in wound healing by clearing apoptotic neutrophils, presenting antigens, and secreting a broad range of cytokines and growth factors, including transforming growth factor-alpha (TGF-α), TGF-beta (TGF-β), basic fibroblast growth factor (bFGF), platelet-derived growth factor (PDGF), and vascular endothelial growth factor (VEGF) [15,17]. These mediators promote the activation and recruitment of endothelial cells, fibroblasts, and keratinocytes, thereby facilitating cell proliferation, ECM synthesis, and angiogenesis. Impaired macrophage function at the wound site can delay wound closure and the resolution of inflammation, particularly in diabetic wounds [4,15]. Lymphocytes are the last immune cells to arrive at the wound site, where they initiate antigen-specific immune responses against pathogens and foreign materials: B-lymphocytes contribute through antibody production, and T-lymphocytes act via cytokine secretion and the induction of cytolytic activity. Lymphocyte-driven inflammation is subsequently resolved through apoptosis following the local production of interferon-γ (IFN-γ) and tumor necrosis factor-α (TNF-α) [18].

The subsequent proliferative phase overlaps with the inflammatory phase and is characterized by angiogenesis, synthesis of ECM components, and re-epithelialization [4,13,15,19]. In response to hypoxia and growth factors such as PDGF and VEGF, angiogenesis is initiated, leading to the formation of new blood vessels during tissue repair [4,19]. During angiogenesis, fibroblasts proliferate and synthesize ECM components, including type III collagen, fibronectin, hyaluronic acid (HA), and proteoglycans. Afterward, fibroblasts differentiate into myofibroblasts, promoting wound contraction and thereby reducing wound surface area, enhancing the mechanical strength of the surrounding tissue [4,13,15,19]. Re-epithelialization refers to the restoration of the epithelial surface through the migration and proliferation of keratinocytes from the wound edges to cover the defect [5,12,15]. Following re-establishment of the epithelial layer, keratinocytes and fibroblasts secrete type IV collagen to form the basement membrane. Keratinocytes then proliferate and undergo morphological changes, enabling complete re-epithelialization and restoration of the epidermal barrier, which protects against infection and prevents excessive moisture loss [15].

The remodeling phase is characterized by the regression of neovascularization and the gradual replacement of type III collagen with the mechanically stronger type I collagen. As remodeling progresses, myofibroblasts play a crucial role in the selective degradation of ECM components through the production of matrix metalloproteinases (MMPs) [4,12,15,19]. Macrophages also contribute to this process by degrading excess ECM and phagocytosing cellular debris [4]. Wound remodeling is a prolonged process that may persist for up to one year following injury [4,15]. Ultimately, scar formation represents the final outcome of wound healing [15].

In summary, cutaneous wound healing is a complex, dynamic, and multistage biological process that requires the coordinated activity of numerous cell types, growth factors, and cytokines. Disruption or dysregulation of any of these components may arrest or impair the healing cascade at various stages, ultimately resulting in the development of chronic, non-healing wounds [15,20].

Diabetic foot ulcers

Diabetic foot is a significant complication of diabetes. As the disease progresses, ulcers develop on the feet, which can be difficult to treat. These ulcers may become extensive, progressive, and can involve increasingly proximal areas of the lower limb [21,22]. Chronic hyperglycemia, advancing neuropathy, immunological dysregulation, chronic inflammation, and microcirculation disorders in the distal parts of the lower limbs result in inadequate oxygen and nutrient supply to the tissues, gradually leading to wound formation [22,23]. The healing of DFUs is limited by multiple factors. Elevated glucose levels damage the endothelium, ECM proteins, and cells involved in the healing process through the dysregulation of metabolic pathways [23]. Damaged nerve fibers progressively diminish the sensation of pain, which is associated with repeated microtraumas that alter the biomechanics of the foot. This alteration modifies the areas of increased pressure, ultimately leading to foot deformities [24,25]. Additionally, autonomic neuropathy induces microcirculation disorders, resulting in insufficient oxygen supply to the tissues, impaired delivery of medications and immune system components, and dry skin [21-23].

The localization of DFUs poses challenges in maintaining wound sterility and hygiene, which, combined with immunological disorders, promotes the development of infections. Infections caused by pathogens such as *Staphylococcus aureus* and *Pseudomonas aeruginosa* and anaerobic bacteria often lead to biofilm formation [13,26]. Additional complicating factors in the treatment of DFUs include the coexistence of thromboembolic disease, circulatory failure, malnutrition, obesity, smoking, inadequate glycemic control, and limited physical activity [13,27].

Burn wounds

BWs resulting from thermal factors present significant challenges in treatment. The healing prognosis is closely related to the severity of the thermal injury, which is classified into four degrees of burns in the literature.

First-degree (I°) burns are superficial, affecting only the epidermis. The skin appears reddened and blanches under pressure, with no blister formation. These burns typically heal without leaving scars [28,29].

Second-degree (II°) burns involve partial thickness of the skin, extending into the dermis, and are divided into two subtypes: IIA and IIB. Subtype IIA (II°A) is characterized by the formation of blisters filled with serous fluid, a well-perfused and highly painful wound bed, preserved sensation, and a slight tendency toward scarring [28-30]. Subtype IIB (II°B) presents additional partial necrosis of the epidermis, with the wound base being less perfused than in subtype IIA. This subtype may exhibit sensory disorders in the burn area, potentially reducing pain levels. It is highly likely to result in hypertrophic scars and often necessitates surgical intervention [28,30].

Third-degree (III°) burns involve the full thickness of the skin, with necrosis present and destruction of the nervous fibers, leading to a complete loss of pain sensation. Only the wound edges remain painful and capable of healing due to their vascularization and perfusion. This type of burn requires surgical treatment and skin transplantation [28,29].

Fourth-degree (IV°) burns are deep injuries that encompass both second- and third-degree burns, potentially penetrating deeper tissues such as subcutaneous fat, muscles, or even bones. These burns generally necessitate surgical intervention [28].

Within BWs, three zones can be identified: the zone of coagulation (necrosis), the zone of stasis (damaged blood vessels), and the zone of hyperemia (a temporary increase in blood flow due to dilated blood vessels). The zone of stasis is at constant risk of secondary necrosis, which can exacerbate the depth of the wounds and complicate the healing process. An additional complication is the occurrence of hypovolemia due to massive effusion, which exacerbates hypoperfusion, promotes ischemia, and leads to inadequate delivery of nutrients, oxygen, and elements of the immune system necessary for proper healing. Necrotic areas are particularly susceptible to colonization by microorganisms such as *S. aureus* and *P. aeruginosa*, which can form biofilms. The presence of these biofilms may lead to infections both locally and systemically [28-31].

Probiotics in regenerative processes

The topical application of probiotics serves as an effective wound therapy through four key mechanisms. First, their primary antimicrobial action is mediated by the secretion of antimicrobial agents, such as hydrogen peroxide and bacteriocins, which also influence biofilm formation [1-3,32,33]. Second, probiotics interact with the immune system during the inflammatory phase of wound healing, modulating responses such as cytokine levels, which are beneficial when present early in the healing process [4,5,32-34]. Third, probiotics produce organic acids that lower the pH of the wound environment, thereby accelerating healing. Additionally, they can adhere to human keratinocytes and fibroblasts, establishing a protective barrier against external pathogens [6,7,32,33,35]. Fourth, specific probiotic strains stimulate the production of growth factors and ECM components, which support the migration and proliferation of skin cells essential for tissue regeneration and wound closure [32,33]. The probiotic microorganisms listed below were selected based on research sources related to clinical treatments and studies conducted on various models. These microorganisms were applied directly to wounds, demonstrating tangible pro-healing effects. Delivery methods and usage guidelines are also included. The most widely discussed microorganisms utilized as probiotics in wound management are summarized in Table 1.

**Table 1:** Probiotic microorganisms used in wound management.

Species	Strains	References
Lactobacillus plantarum	ATCC 8014, ATCC 10241, USM8613, NCIMB 8826	[32,36-39]
Lactobacillus reuteri	DSM 17938, BNCC 192190, modified strains producing CXCL12	[35,40-43]
Lactobacillus rhamnosus	IBRC-M 11409	[44-46]
Lactobacillus paracasei	TYM202	[47]
Bacillus velezensis	M76T11B	[47]

*Lactobacillus* species

L. plantarum

When applied locally to II-III° BWs in humans as gauze, the use of *L. plantarum* cultures demonstrated faster debridement, granulation, and wound closure compared to silver sulfadiazine. Additionally, there was a notable reduction in the presence of *S. aureus* and *P. aeruginosa* [36]. Prophylactic application of alginate films and gels containing viable *L. plantarum* significantly decreased the risk of *P. aeruginosa* infections in a rat model of BW [32,37]. Clinical studies and in vivo trials utilizing gauze infused with both *L. plantarum* culture and its supernatant were conducted to treat chronic wounds infected with *P. aeruginosa*, including DFUs. These studies observed inhibition of quorum-sensing signals, which hindered further biofilm formation [36,38]. In rat excisional wounds, *L. plantarum* (either alone or in combination with *Bifidobacterium longum* and *Lactobacillus rhamnosus*) modulated the levels of interleukins (IL-1β, IL-6), TGF-β, collagen types I and III, and VEGF, thereby enhancing angiogenesis and tissue remodeling [38,39].

Lactobacillus reuteri

Topically applied ointments containing viable *L. reuteri* have been shown to enhance the healing of full-thickness skin wounds in rat models, leading to increased wound contraction, improved collagen distribution, and a significant reduction in inflammation and myeloperoxidase (MPO) levels [40]. Genetically modified strains of *L. reuteri* that produce the chemokine CXCL12, formulated as gel in Gel/L@FeTA, were tested on mouse models, including those with diabetic ulcers, demonstrating a significant acceleration in wound healing through sustained local levels of CXCL12 and lactic acid-mediated enhancement of chemokine bioavailability [35,41-43]. As a component of “living hydrogels” or culture-delivery dressings, *L. reuteri* exhibits strong antibacterial activity against *S. aureus* and *Escherichia coli* due to its continuous production of organic acids and, in some formulations, hydrogen. An alginate gel containing *L. reuteri* and hydrogen nanoparticles (LR&AB@CAH) resulted in reduced ROS and glucose levels, increased angiogenesis, collagen deposition, and M2 macrophage recruitment, thus accelerating wound closure and improving scar quality [35,41].

L. rhamnosus

Multistrain formulations in the form of oleogels containing *L. rhamnosus*, along with *Lactobacillus casei*, *Lactobacillus fermentum*, and *Lactobacillus acidophilus*, enhance cytokine modulation by increasing IL-10 activity while decreasing TNF-α and IL-6 activity. These formulations promote neovascularization, collagen production, fibroblast proliferation, and hair follicle formation in DFUs when compared to controls and antibiotic treatments [44]. A photopolymerized hydrogel (LM-GelMA) containing *L. rhamnosus* GG supernatant, when applied topically to infected wounds, demonstrated strong antibacterial effects and promoted the healing process [45,46].

Other Lactobacillus spp.

Other *Lactobacillus* species, including *L. casei*, *L. fermentum*, *L. acidophilus*, and *Lactobacillus delbrueckii subsp. bulgaricus*, when combined with *L. plantarum* in diabetic ulcers, improved wound size reduction, collagen deposition, vascularization, and modulation of inflammatory cytokines [44,48]. The probiotic *Lactobacillus paracasei* TYM202, formulated as HAEPS@L․sei hydrogel and applied locally to full-thickness skin wounds in rat models, resulted in accelerated healing, enhanced angiogenesis, increased collagen content, and stabilization of the skin microbiota [47].

Probiotic Yeasts: Saccharomyces cerevisiae and Saccharomyces boulardii

*S. cerevisiae*, when incorporated into probiotic collagen hydrogels or scaffolds, has been shown to enhance collagen content, promote angiogenesis, support epithelial regeneration, and improve biomechanical properties of scars compared to collagen alone, as demonstrated in BW models in rats [49]. In a porcine model, *S. boulardii* applied topically to acute skin wounds demonstrated limited benefits when used without a moist carrier, suggesting that the effectiveness of yeast-based wound healing relies on the delivery system [50].

Multistrain Formulas

Kefir, a natural consortium of *Lactobacillus*, *Leuconostoc*, *Acetobacter*, and *Saccharomyces*, when applied as extracts or gels to BW in animal models, resulted in increased fibroblast migration; modulation of IL-1β, TGF-β1, and bFGF expression; facilitated faster re-epithelialization and collagen maturation; and reduced inflammation [8,9,51,52]. Commercial multi-*Lactobacillus* products, such as Bio-K+, when applied topically in ex vivo human skin models, demonstrated the ability to reduce *P. aeruginosa* biofilms and promote dermal fibroblast migration, highlighting the antibiofilm potential of probiotic blends [53].

Strategies of probiotic microorganism usage in the wound healing process 

Among the publications selected for the research, three main strategies can be distinguished in the development of wound dressings based on probiotic cells and components. All the research results discussed below pertain to the use of external dressings and formulations applied directly to the wound.

The first strategy involves utilizing a probiotic strain placed in a carrier, such as hydrogels, with controlled release capabilities, which accelerates wound healing. Hydrogel within the hydrophilic 3D network structures can mimic the ECM and promote cell growth. It is also suggested that the role includes protecting probiotic bacteria from harmful environmental effects. Additionally, hydrogel serves a nutritional function by providing bacteria with essential growth components [5].

The second involves the direct use of whole natural or genetically modified probiotic strains in the wound healing process in forms different from dressing. The third encompasses the use of probiotic-derived products such as cellular elements and metabolites [5].

Dressings containing viable probiotic microorganisms

Alginate Probiotic Microparticles in BW Utilizing L. plantarum

One of the key challenges is creating a delivery vehicle that maintains probiotic viability while interacting favorably with the wound environment. Alginate-based systems are a prominent example of this technology. In situ gelling *L. plantarum* microparticles are produced through spray-drying a chitosan/alginate or hyaluronate feed containing suspended bacteria in a Büchi mini-dryer, with optimization of inlet temperature and polymer concentration utilizing a quality by design (QbD) approach. The resulting powder is free-flowing and, upon contact with wound exudate, rapidly swells into a cohesive hydrogel. This three-dimensional network concentrates viable probiotics at the surface, absorbs exudate, and continuously releases antimicrobial metabolites that suppress *P. aeruginosa* and *S. aureus*, while maintaining the rheological properties and stability required for burn dressings. The formulation accelerated re-epithelialization, with probiotic-treated groups demonstrating the highest level of re-epithelialization after 21 days of therapy. Collagen deposition was significantly enhanced in wounds treated with probiotics, indicative of a more robust ECM essential for healing. Clinically, treated wounds showed immediate improvement, reduced secretions, and minimal necrotic tissue compared to control groups. The overall efficacy of the optimal microparticles was superior to that of the control groups across all parameters in rat models and simulated wound-like fluid. There was a stronger inhibition of *P. aeruginosa* and *S. aureus*, with wound closure time and infection control comparable to or better than silver therapy. Additionally, the microparticles exhibited good stability, with no significant decrease in viable bacteria count after four weeks of storage at 4°C [32].

ProGel: Injectable Hydrogel Containing L. plantarum

Another approach to creating a delivery vehicle is creating an injectable hydrogel. ProGel is an injectable, gelatin-based hydrogel specifically designed to combat multifaceted pathogen wound infections through the encapsulation of *L. plantarum* probiotics. It is produced by dissolving hydrazide-modified gelatin (Gel-ADH) with aldehyde-functionalized polyethylene glycol (DF-PEG) via reversible Schiff-base bonds, forming hydrazones at neutral pH. The probiotic *L. plantarum* is premixed with the DF-PEG solution prior to cross-linking. The hydrogel is applied using a dual-syringe system, facilitating rapid in situ gelation (approximately one minute) and ensuring seamless conformation to complex wound geometries. Rheological assessments demonstrate a solid-like network that provides self-healing and shape adaptability. Viability assays indicate that ≥80% of the bacteria remain active, with no detectable leakage in phosphate-buffered saline (PBS) or agar. The mesh size of the hydrogel is significantly smaller than that of *L. plantarum*, effectively restricting the escape of probiotics while allowing the diffusion of nutrients and antimicrobial agents produced by the probiotics, such as organic acids and plantaricins. The hydrogel swells to approximately 160% within 24 hours across various pH levels (6-8), retaining moisture and absorbing wound exudate, which promotes healing.

ProGel exhibits high cytocompatibility with human dermal fibroblasts (HDFs), indicating its safety for direct contact with human cells. Additionally, it is hemocompatible, meaning it does not cause significant hemolysis, which is essential for materials used in wounds that may interact with blood. Moreover, it demonstrates broad-spectrum antimicrobial efficacy against common wound pathogens, notably *P. aeruginosa*, *S. aureus*, and *Candida albicans*. It prevents biofilm formation on human skin, while ex vivo human skin experiments show effective biofilm prevention on wounds. Histological analyses confirm an intact dermis with minimal inflammation [54].

PU/PRGF/Gelatin/PU With L. plantarum (Multilayer Scaffold)

A study conducted by Shahghasempour et al. introduced a bioactive multilayer electrospun scaffold (PU/PRGF/gelatin/PU) that incorporates *L. plantarum* and platelet-rich growth factor (PRGF) to enhance wound healing and prevent infections. This scaffold is designed to mimic the properties of the ECM. The PU/PRGF/gelatin/PU scaffold consists of four layers, fabricated by melt-electrospinning polyurethane (PU) to create outer meshes, electrospinning a central gelatin layer loaded with PRGF, and subsequently seeding the surface with fibroblasts, human adipose-derived mesenchymal stem cells (hAMSCs), and *L. plantarum* ATCC 8014. This highly porous and mechanically stable composite functions as a bioactive dermal substitute: PRGF serves as a sustained reservoir of growth factors, the hydrophilic gelatin layer facilitates cell colonization, and the surface probiotic biofilm secretes metabolites such as hydrogen peroxide, lactic acid, and bacteriocins, contributing to its antibacterial properties. These properties significantly reduce the presence of pathogens, including *S. aureus*, *E. coli*, *P. aeruginosa*, *Salmonella* spp., and *Klebsiella* spp. Scaffolds containing *L. plantarum* demonstrate over 70% reductions in CFU compared to probiotic-free controls. PRGF comprises growth factors such as PDGF, TGF, VEGF, and epidermal growth factor (EGF), which promote mitosis and angiogenesis and enhance fibroblast proliferation. The electrospraying of PRGF increased the scaffold's hydrophilicity, thereby improving cell adhesion. Overall, this scaffold significantly enhanced the viability and proliferation of cocultured hAMSCs and increased fibroblast migration and proliferation, effectively supporting cell attachment, while controlling infection [55].

Gauze Containing L. plantarum Culture in II/III° Burns

Not all delivery approaches involve complicated delivery vehicle systems. Peral et al. designed a study in which researchers utilized whole cultures of the *L. plantarum* ATCC10241 strain grown in MRS (de Man-Rogosa-Sharpe) broth, which were then spread on sterile gauze. The study involved a total of 34 patients, comprising 14 individuals diagnosed with moderately controlled type 2 diabetes mellitus and 20 patients with venous ulcers. A once-daily topical use of this dressing was administered for 10 days without the use of additional antimicrobials. After 10 days of treatment, the bacterial load in the wounds of diabetic patients significantly decreased, effectively eliminating biofilm-producing bacteria such as *S. aureus* and *P. aeruginosa*. *L. plantarum* has been shown to antagonize *P. aeruginosa* in vitro by inhibiting quorum-sensing signals, biofilm formation, and virulence factors. In vivo, it decreases infective capacity and enhances the phagocytic activity of polymorphonuclear neutrophils (PMNs).

The proposed mechanism suggests that treatment with *L. plantarum* regulates IL-8 levels and modulates the entry and activity of newly arrived peripheral blood PMNs (PBPMNs) to the ulcer bed. The acidic, living biofilm facilitates debridement and decreases apoptosis and necrosis of neutrophils, promoting the presence of fresh PMNs, macrophages, fibroblasts, and endothelial cells. This leads to a decrease in bacterial load, less debris, and a diminished inflammatory response, ultimately supporting tissue repair processes. There were no significant differences in wound healing or bacterial load reduction between the diabetic and non-diabetic groups [38].

Alginate Films Containing L. plantarum in BW

Alginate films containing *L. plantarum* are produced by mixing log-phase cultures with sodium alginate, followed by casting, cross-linking in CaCl₂, and freeze-drying to create thin calcium-alginate sheets. Upon rehydration over BW, these films absorb exudate and release acids and bacteriocins, resulting in a reduction of *P. aeruginosa* without systemic translocation. Moreover, they retain viable probiotics for a duration of at least six months when stored at 4°C [37].

GelNBSH-L Photopolymerized Hydrogel: L. reuteri

This dressing utilizes the antimicrobial and health-promoting properties of *L. reuteri*, which releases secretions such as reuterin, ethanol, and organic acids. GelNBSH-L is constructed by functionalizing gelatin with norbornene (GelNB) and sulfhydryl groups (GelSH), resulting in the generation of monodisperse alginate microspheres (AlgMPs) loaded with *L. reuteri* through microfluids. The mixture is then photopolymerized in less than 1 s under UV light exposure, yielding an injectable, self-supporting GelNBSH-L hydrogel characterized by a 3D interconnecting porous structure. This double encapsulation system protects *L. reuteri* from the harsh wound environment, sustains its viability, and limits leakage. Full-thickness excisional wounds were created on mice and inoculated with pathogens. The wounds were subsequently covered with GelNBSH-L hydrogel and in situ polymerized. The dressing achieved over 90% sterilization against *S. aureus* and *E. coli* in vitro. In vivo, it demonstrated accelerated wound healing, stimulated vascularization and denser collagen deposition, and promoted re-epithelialization and granulation. Additionally, there was a decrease in the expression of TNF-α, indicating significant mitigation of inflammation, all while maintaining high probiotic viability and biosafety [42].

*Gel/L@FeTA Hydrogel: CXCL12-Producing L. reuteri Coated by a Fe-Tannin Comple*x

This dressing utilizes modified *L. reuteri* strains producing chemokine CXCL12, also known as stromal cell-derived factor 1 (SDF-1). CXCL12 is a critical homeostatic chemokine that regulates cell migration, embryogenesis, hematopoiesis, and angiogenesis [5]. Gel/L@FeTA is created by coating *L. reuteri* with alternating layers of tannic acid/Fe³⁺ (FeTA) to form *L. reuteri*@FeTA, then dispersed in a carboxymethyl-chitosan/oxidized-hyaluronan solution that undergoes gelation via Schiff-base reactions. The FeTA shell adsorbs gentamicin, penicillin, and cephalosporins, preventing their direct contact with the probiotic, while the hydrogel maintains water absorption and mechanical properties, retaining its antimicrobial efficacy. This technology addresses the challenge of proteolytic degradation in wounds by enabling localized chemokine production and reducing degradation. The *Lactobacilli* produce lactic acid, which lowers the local wound pH by 0.2-0.5. This acidic environment inhibits the peptidase CD26, preventing the degradation and inactivation of CXCL12, thereby increasing its bioavailability. Treatment results in enhanced proliferation of dermal cells and increased density of macrophages, thereby accelerating the healing process. In hyperglycemic mice, the treatment normalized the wound-induced increase in blood flow, which was previously absent. Initial safety studies demonstrated that the effects are localized to the wound site, with no detectable systemic CXCL12 or bacteria in circulation. In vitro, it preserves probiotic growth and lactic acid release under antibiotic exposure and reduces biofilms of *S. aureus* and *E. coli*; in vivo, it accelerates closure and improves collagen and adnexal regeneration, with or without concurrent systemic antibiotics [41].

*“Living Bacterial Hydrogel” With L. reuteri (LRHA)​​​​​*​​

Researchers developed a living bacterial hydrogel (LRHA) aimed at accelerating the healing of infected wounds by encapsulation of *L. reuteri* within gelatin microspheres through emulsion polymerization. These microspheres were subsequently immobilized in a methacrylated HA hydrogel network using photopolymerization, resulting in the formation of LRHA. This innovative process enables the in situ generation of the hydrogel dressing at the wound site. The hydrogel scaffold containing encapsulated *L. reuteri* protects the bacteria from immune system attack and prevents leakage. Plate and broth ensure that the bacteria do not escape but instead proliferate within the microspheres. They secrete lactic acid and reuterin, which diffuse out to inhibit the growth of pathogens, including *S. aureus*, *E. coli*, and *Salmonella* spp. In vivo experiments conducted using a mouse model of infected full-thickness wounds demonstrated several physiological benefits. Treatment with LRHA resulted in significantly reduced inflammatory infiltrates, characterized by a smaller number of neutrophils and notably less bacterial CFU observed in the infected skin by day 7. Additionally, LRHA treatment enhanced collagen deposition, promoted neovascularization, decreased inflammatory infiltrates, and accelerated the regeneration of epidermal tissue, resulting in a thinner, more physiological epithelial layer compared to the control and HA groups. Complete wound closure was achieved by day 10 with LRHA, whereas the HA group exhibited closure by day 13 and the control group by day 15. The material demonstrated good biocompatibility, with no significant differences in inflammatory cytokines or abnormal defects observed in the major organs of the treated mice [43].

LR&AB@CAH: Alginate Gel Containing L. reuteri and H₂ Nanoparticles

The study by Wang et al. presented a probiotic active gel, LR&AB@CAH, specifically designed for the treatment of DFUs by effectively addressing elevated glucose levels and oxidative stress. LR&AB@CAH is created by dissolving 3% sodium alginate, incorporating AB-loaded mesoporous silica nanoparticles and *L. reuteri* BNCC192190, followed by casting the mixture into molds and cross-linking with 10% CaCl₂ to form calcium-alginate hydrogels. This gel operates through a cascade mechanism. *L. reuteri* metabolizes excess glucose in the wound, which reduces local hyperglycemia and produces lactic acid. The lactic acid subsequently lowers the pH of the wound, activating acid-responsive aminoborane-loaded mesoporous silica nanoparticles (AB@MSN) to generate hydrogen. This hydrogen functions as an antioxidant, selectively scavenging harmful hydroxyl and nitrite radicals, thereby neutralizing excess ROS. This dual action targets both the source of ROS production (high glucose) and the existing ROS, effectively reducing oxidative stress and inflammation, which fosters a more conducive environment for wound healing.

In full-thickness diabetic mouse wounds, the application of LR&AB@CAH every two days resulted in the fastest wound closure kinetics, significantly decreasing tissue glucose and ROS levels compared to control groups. Additionally, it reduced markers of oxidative damage and improved collagen alignment and re-epithelialization relative to controls. In vitro studies demonstrated that the gel supports glucose diffusion and consumption (as monitored by enzymatic assays), facilitates progressive lactic acid accumulation, and triggers hydrogen release from AB@MSN under acidic conditions. This leads to decreased levels of ROS and lipid peroxidation markers, alongside increased activity of antioxidant enzymes. In vivo, LR&AB@CAH significantly accelerated wound closure in diabetic mice, with noticeable improvements observed by day 14. Histological analysis showed reduced inflammatory cell infiltration, enhanced restoration of epidermal and hair follicle structures, and a more organized arrangement of collagen fibers. The gel also significantly lowered glucose levels in the wound bed and enhanced antioxidant enzyme activity, while concurrently reducing levels of oxidative substances and inflammatory markers. Transcriptomic analysis revealed that LR&AB@CAH regulated 112 core genes, primarily enriched in pathways associated with inflammatory responses and oxidative stress, facilitating wound normalization. The study concluded that LR&AB@CAH offers a comprehensive and effective approach for treating DFUs by simultaneously consuming glucose and scavenging ROS. This artificial-natural composite hydrogel exhibited good biocompatibility and significant therapeutic efficacy in promoting diabetic wound healing in a mouse model [35].

*HAEPS@L*․*sei: Extracellular Polymeric Substances + L. paracasei-Based Hydrogel*

A multi-cross-linked probiotic hydrogel, referred to as HAEPS@L․sei gel, was developed to enhance wound healing by preserving a stable skin microbiota and mitigating inflammation. This hydrogel was formulated using dopamine-modified extracellular polysaccharide (EPS-M76) derived from *Bacillus velezensis* M76T11B, HA methacrylate (HAMA), and live *L. paracasei* TYM202. The dopamine-modified EPS-M76 (DAEPS) and HAMA established an initial cross-linking network through hydrogen bonding, followed by secondary covalent cross-linking induced by blue light irradiation. EPS-M76 served as a prebiotic, promoting the proliferation and metabolic activity of *L. paracasei* TYM202, which in turn led to increased secretion of lactic acid and acetic acid.

A full-thickness skin injury model in male rats was employed to assess the wound healing efficacy of the probiotic hydrogels. These hydrogels significantly reduced inflammation by facilitating the transition of macrophages from the pro-inflammatory M1 phenotype to the anti-inflammatory M2 phenotype, resulting in elevated levels of anti-inflammatory factors such as IL-10, while decreasing pro-inflammatory factors like TNF-α. The hydrogel also promoted angiogenesis and enhanced collagen deposition during the remodeling phase, thereby contributing to tissue regeneration. Additionally, it helped maintain a slightly acidic wound environment, which is essential for optimal macrophage response, fibroblast proliferation, angiogenesis, and collagen synthesis. The HAEPS@L․sei hydrogel exhibited superior wound healing capabilities. By day 14, the rate of wound closure in the treated rats was significantly greater than that observed in the control groups. The hydrogel demonstrated excellent biocompatibility and non-hemolytic properties and supported cell proliferation and migration. Furthermore, it effectively maintained a stable skin microbiota structure while successfully repelling pathogenic bacteria without compromising beneficial microbial communities. The organic acids secreted by the hydrogel inhibited the growth of pathogenic bacteria, including *S. aureus* and *E. coli*, demonstrating antibacterial activity against these pathogens [47].

Collagen Hydrogel/Scaffold + S. cerevisiae in BW Treatment

The researchers examined the simultaneous application of a probiotic microorganism and a collagen hydrogel-scaffold (CH-S) to enhance BW healing in a rat model. The hydrogel-scaffold, termed *S. cerevisiae* with collagen hydrogel-scaffold (CH-S-S), begins with a porous collagen sponge created through freeze-drying. This sponge is then infiltrated with a neutralized CH-S and supplemented with a suspension of *S. cerevisiae* prior to gelation.

Six different BW treatment methods were evaluated: silver sulfadiazine (SSD) served as a positive control, representing a conventional wound dressing approach; untreated wounds acted as the negative control; a suspension of *S. cerevisiae* (S.C.) was locally injected into select wounds; a collagen scaffold (CS) was applied as a monophase treatment; the collagen hydrogel-scaffold (CH-S) represented a biphasic treatment, combining collagen hydrogel with a scaffold; and *S. cerevisiae* with collagen hydrogel-scaffold (CH-S-S) was a tri-phasic treatment where *S. cerevisiae* suspension was locally injected, followed by coverage with the collagen hydrogel-scaffold. Rats were euthanized at 12 and 22 days post-injury for further assessments.

The CH-S-S group exhibited the most favorable healing outcomes, achieving nearly complete wound closure by 12 days post-injury. This group displayed significantly increased collagen content, greater ultimate load, and increased stiffness compared to untreated wounds. The combination treatment of *S. cerevisiae* and CH-S-S resulted in a substantial reduction of inflammatory cells, complete epithelial layer formation with rete ridges, and enhanced biomechanical performance, while also minimizing scar size. Further analysis indicated elevated expression levels of collagen type I and TGF-β, suggesting a higher maturation rate in the wound area. Collagen scaffolds facilitate the deposition and organization of new collagen fibers and granulation tissue. Hydrogels, serving as wet dressings, provide essential moisture to the wound bed, regulate scarring, and promote fibroblast proliferation. The hydrogel in the CH-S-S treatment was crucial for activating *S. cerevisiae*, which might otherwise remain inactive due to wound dehydration. Probiotic microorganisms such as *S. cerevisiae* are thought to help prevent wound infections by occupying niches that could be colonized by pathogens or by acidifying the wound environment, thereby inhibiting bacterial growth. Additionally, they presented with good biocompatibility [49]. While these hydrogel-based systems show promise, they differ significantly in their formulation and manufacturing complexity.

Application of viable probiotic microorganisms in wound treatment in forms other than dressings

Direct Application of Bacterial Suspension to the Wound

Due to the high complexity of hydrogel-based delivery vehicles, simpler methods have also been evaluated. This method is based on the direct application to the wound of a liquid medium containing microorganisms suspended at a defined concentration. Suspensions containing *L. plantarum* have been observed to increase hydroxyproline levels, an indirect marker of collagen synthesis in tissue and thus an indicator of reparative processes, i.e., wound healing [44]. Peral et al., in a cohort of 80 patients, demonstrated that the direct application of *L. plantarum* to the wound promotes autolytic debridement, granulation, and healing of II° and III° BW. Additionally, it was observed that this simple regimen reduced the presence of *S. aureus* and *P. aeruginosa*. The effectiveness of this approach was assessed as comparable to dressings based on silver sulfadiazine [36].

Probiotic Ointments

Khodaii et al. studied the effects of probiotic *L. reuteri* DSM17938 ointment usage on full-thickness sink wounds on rat models. Research on animal models has shown that probiotic ointments reduced inflammation, accelerated epithelialization, and deposited collagen. Additionally, lowered MPO activity was observed, which led to reduced oxidative damage [40].

Oleogel-Based Formulation

Another example of topical use of probiotic bacteria is suspending several species of *Lactobacilli* in oleogel-based medium as described by Karimi et al. Oil-based gel containing viable *L. rhamnosus*, *L. casei*, *L. fermentum*, and *L. acidophilus* has been applied to skin wounds on rat models. Compared with topical tetracycline, probiotic-treated ulcers showed a greater percentage reduction in area, higher hydroxyproline content, more fibroblasts and hair follicles, denser and better organized collagen, and richer neovascularization, implying that continuous local metabolic activity of the bacteria can outperform a conventional topical antibiotic in tissue repair [44].

Direct Application of Probiotic Yeasts

*S. cerevisiae* suspensions have been spread onto or injected into deep partial-thickness burns in rats. As a standalone treatment, yeast modestly improved epithelialization and infection control, but the most pronounced benefit occurred when it was combined with a collagen-hydrogel scaffold. In that tri-phasic dressing, *S. cerevisiae* plus collagen scaffold significantly increased collagen content and tensile strength, produced thicker, more organized neo-epidermis and dermis, and shortened healing times compared with silver sulfadiazine or collagen alone, suggesting synergistic effects between the probiotic metabolism and the biomaterial matrix [49].

Biohybrid Microneedles With Probiotic Yeast

Another method was the use of microneedles as proposed by He et al. The main problem with standard treatments was identified: poor tissue penetration and the need for repeated application. A potential solution was recognized in dissolving microneedles made from biocompatible materials such as HA and collagen-derived peptides. These offer minimally invasive intradermal delivery of live *S. cerevisiae*. As a metabolically active component, the yeast continuously releases ethanol and β-glucan, providing combined antibacterial and immunomodulatory effects. In diabetic wound mouse models with infection, a single application of yeast-loaded microneedles improved bacterial control and accelerated wound closure. This approach illustrates the potential of biohybrid microneedle systems as a sustained, multifunctional strategy for difficult-to-heal wounds [56].

Fermented Probiotic Products: Topical Application

Kefir is a natural, complex consortium comprising lactic acid bacteria, acetic acid bacteria, and yeasts. Kefir-based gels have been administered directly to rodent BW models and cutaneous defects over a duration of approximately three weeks. These fermented formulations reduced inflammatory markers and facilitated a more rapid formation and maturation of tissue through enhanced proliferation and migration of HDF cells. Additionally, they promoted enhanced re-epithelialization, increased collagen deposition, augmented neovascularization, and improved the overall quality of scar tissue. The outcomes observed were superior to those in untreated controls or silver sulfadiazine-treated [49,51].

Usage of postbiotics in topical wound treatment

Cell-Free Supernatants From L. plantarum

Cell-free supernatants derived from *L. plantarum* cultures are abundant in a variety of bioactive compounds, including high concentrations of D- and L-lactic acid, hydrogen peroxide, phenolic compounds, and essential cations such as calcium (Ca), magnesium (Mg), and potassium (K), as well as a variety of enzymes and small organic molecules like barbituric acid derivatives. The therapeutic mechanism of these supernatants is multifaceted. Lactic acid directly stimulates the production of endothelial growth factors and promotes angiogenesis, thereby enhancing the vascularization of the wound bed [57]. Other components within the supernatant actively disrupt the quorum-sensing mechanisms of *P. aeruginosa*, inhibiting elastase activity and effectively reducing both biofilm formation and the expression of virulence factors by this pathogen [39]. In the context of chronic leg ulcers, these preparations modulate the production of IL-8 by neutrophils present in the wound and decrease the apoptosis and necrosis of polymorphonuclear cells, which collectively lead to more efficient wound debridement and resolution of inflammation. These supernatants are utilized as a topical liquid, either instilled directly into the wound or used to impregnate dressings for treating infected chronic ulcers and burns. The observed effects include a significant reduction in pathogenic burden and necrotic tissue, a decrease in the size of the wound area, enhanced debridement and granulation tissue formation, and effective disruption of *P. aeruginosa* biofilms, all while demonstrating no cytotoxicity when compared to conventional antiseptics [57].

Protein-Rich Postbiotic Fractions From L. plantarum USM8613

Protein-rich postbiotic fractions derived from *L. plantarum* USM8613 consist of a concentrated blend of proteins, including plantaricins such as plantaricin EF and plantaricin JK. The primary mechanism of action involves inhibiting the synthesis of staphyloxanthin in *S. aureus*, a crucial antioxidant pigment for the pathogen, making it more vulnerable to oxidative stress. These fractions also effectively reduce bacterial cell counts and decrease biofilm thickness. Furthermore, they strongly induce the expression of β-defensin and trigger favorable shifts in cytokine profiles within the wound environment. This includes an increase in anti-inflammatory and pro-healing cytokines like IL-4, IL-6, and TGF-β, while simultaneously reducing pro-inflammatory mediators such as TNF-α and MMPs. These protein-rich fractions are formulated into topical ointments or gels and applied to infected wounds, as demonstrated in rat and porcine skin models. The resulting therapeutic effects encompass the suppression of *S. aureus* infection and the development of thinner biofilms, accelerate re-epithelialization, and improve histological parameters of wound repair [58].

Lactobacillus Biofilm Derivatives for Diabetic Wounds

*Lactobacillus* biofilm derivatives represent a unique class of postbiotic agents, meticulously prepared by applying ultrasound and filtration techniques to *Lactobacillus *biofilms. This process yields a complex ECM material, rich in polysaccharides, proteins, lipids, and deoxyribonucleic acid, but crucially devoid of any viable bacterial cells. The therapeutic mechanism of these derivatives centers on their ability to reprogram macrophage metabolism. They achieve this by inhibiting the Janus kinase-signal transducer and activator of transcription 1 (JAK-STAT1) signaling pathway, facilitating a phenotypic shift in macrophages from a pro-inflammatory M1 state to a reparative M2 state. This metabolic re-orchestration alleviates local inflammation, promotes neovascularization, and actively supports overall tissue regeneration. These derivatives are administered topically to full-thickness diabetic mouse wounds, either as a bacteria-free liquid or as an integrated component of wound dressings. Beneficial effects observed include a significant reduction in inflammatory cytokines, enhanced granulation tissue formation, improved blood vessel development, and faster closure of diabetic wounds, all without the inherent risk of bacteremia associated with live bacterial therapies [59].

Lactobacillus Membrane Vesicles in Bacteriomimetic Hydrogels

Bacteriomimetic hydrogels incorporate nanoscale membrane vesicles naturally released by *L. plantarum* and *L. casei*. These vesicles are characterized by their rich content of surface and cargo proteins, which can vary depending on the bacterial strain and the specific culture conditions used for their production. The core mechanism involves interaction with primary human immune cells, where they have been shown to decrease levels of TNF-α while simultaneously increasing levels of IL-10. This leads to an increased IL-10/TNF-α ratio, exerting a net anti-inflammatory effect, especially when bacteria are cultured anaerobically. For application, these membrane vesicles are covalently coupled to synthetic microparticles, forming "bacteriomimetic" beads. These beads are then embedded within a topical hydrogel matrix, which is applied to full-thickness wounds created on mouse tails. The therapeutic outcomes include accelerated wound closure, support for keratinocyte migration, enhanced re-epithelialization, development of a thicker dermal layer, increased revascularization, reduction in the number of neutrophils, and diminished scarring [60].

Angiogenic Supernatants From Lactobacillus Cultures

Complex supernatants derived from *Lactobacillus *cultures contain multiple unidentified bioactive molecules in addition to lactic acid. These supernatants exhibit potent pro-inflammatory and angiogenic activities, influencing tissue repair and immune responses. *Lactobacillus *supernatants were administered subcutaneously into the ear lobes of mice. The results indicated stimulation of inflammation, an increase in TNF-α level, and the formation of new blood vessels, with strong immunostaining observed for VEGF receptors. This process promotes the growth of macrophage immune cells and enhances lymphocyte activity. Importantly, these effects are not due to bacterial lipopolysaccharide, a common bacterial toxin, as no particular substance was identified. Subcutaneous injection into rodent ears stimulated angiogenesis; however, this angiogenic effect was not observed in skin wounds, possibly due to differences in the local environment and an excessive promotion of inflammatory response [61].

Postbiotic Creams Based on Fermented Probiotic Products: Kefir

Cold cream is a historically recognized water-in-oil emulsion composed of natural ingredients that create lipid-like coatings and soothe the skin [62]. The natural constituents of kefir microorganisms include *L. fermentum*, *L. reuteri*, and *Bacillus subtilis natto*, whose supernatants were incorporated into a cold cream formulation devoid of viable microbes. In full-thickness skin wound models in rats, these postbiotic creams were applied topically once or twice daily. Observations revealed significantly elevated hydroxyproline levels, accompanied by thicker and more cellular granulation tissue, increased fibroblast density, and expedited epithelial coverage. Histological analysis demonstrated improved collagen deposition and fewer necrotic areas. Further analysis suggested that secreted bacteriocins, organic acids, and small immunomodulatory molecules derived from these probiotic species mitigate excessive neutrophil infiltration and downregulate inflammatory cascades, while upregulating TGF-β and other growth factors. This mechanism appears to shorten the inflammatory phase while accelerating proliferation and remodeling without the risk of translocation of live bacteria. Compared to base cream and untreated control, postbiotic formulations exhibited significantly enhanced healing outcomes [44].

## Conclusions

This review represents an early effort to organize the current body of knowledge regarding the use of probiotics in the treatment of chronic wounds, such as diabetic ulcers and burn injuries. Wound management, despite continuous advances in dressing materials, remains a challenge for both clinicians and healthcare systems. The high costs and prevalence of chronic wounds and burn injuries make their treatment - particularly strategies that are efficient, effective, and capable of reducing complications such as infection - a substantial clinical and economic burden. In recent years, interest in probiotics has broadened to include their potential application in wound care, including incorporation into dressing materials. Although the modes of application of probiotic microorganisms, fungi, and their derivatives - containing both probiotic and postbiotic approaches - vary considerably, many studies suggest a positive influence on wound healing-related processes.

Current literature places particular emphasis on the therapeutic potential of *L. plantarum* as a probiotic strain in wound management. This microorganism may enhance wound healing not only by inhibiting the formation of biofilms composed of pathogenic bacterial strains but also by directly modulating key cellular and molecular mechanisms involved in tissue repair. Despite the availability of data describing various methods of probiotic delivery and incorporation into wound dressings, direct comparisons of their relative efficacy are currently not feasible, as no head-to-head studies have yet been conducted. In our view, addressing this gap represents a critical next step for future research. Additionally, heterogeneity in strain selection, formulation, and outcome measures further limits the comparability of existing studies. However, much of the available evidence derives from in vitro and animal models. Robust clinical data in human populations remain limited, and the therapeutic value of probiotic-based wound therapies has yet to be clearly established. At present, such strategies should be regarded as investigational, warranting further well-designed experimental and clinical studies to clarify their efficacy and safety. Future research must prioritize well-designed randomized controlled trials in human subjects to validate the promising results seen in animal models and to determine optimal dosing, formulation, and safety profiles for clinical use.
